# Stable clinical risk prediction against distribution shift in electronic health records

**DOI:** 10.1016/j.patter.2023.100828

**Published:** 2023-08-22

**Authors:** Seungyeon Lee, Changchang Yin, Ping Zhang

**Affiliations:** 1 Department of Computer Science and Engineering, The Ohio State University, Columbus, OH 43210, USA; 2 Department of Biomedical Informatics, The Ohio State University, Columbus, OH 43210, USA

**Keywords:** deep learning, stable learning, clinical risk prediction, EHR study, distribution shift, patient representation learning, sample reweighting

## Abstract

The availability of large-scale electronic health record datasets has led to the development of artificial intelligence (AI) methods for clinical risk prediction that help improve patient care. However, existing studies have shown that AI models suffer from severe performance decay after several years of deployment, which might be caused by various temporal dataset shifts. When the shift occurs, we have access to large-scale pre-shift data and small-scale post-shift data that are not enough to train new models in the post-shift environment. In this study, we propose a new method to address the issue. We reweight patients from the pre-shift environment to mitigate the distribution shift between pre- and post-shift environments. Moreover, we adopt a Kullback-Leibler divergence loss to force the models to learn similar patient representations in pre- and post-shift environments. Our experimental results show that our model efficiently mitigates temporal shifts, improving prediction performance.

## Highlights

• We study temporal shifts within EHRs and the performance decay caused by the shifts
• We propose a method that reweights patients to reduce the temporal data shift
• The proposed method can efficiently leverage the data from different environments

## The bigger picture

Artificial intelligence (AI) methods that rely on large electronic health record datasets have proved effective at predicting different kinds of “clinical risk,” the likelihood of a disease or adverse outcome given past clinical history. While these methods can help improve patient care, there are still substantial obstacles to their widespread use. In particular, studies have shown that these kinds of AI models perform more poorly after several years of deployment due to shifts in the underlying data distributions, caused by, for example, changes in patient populations or medical practice. Here, the authors propose a method that reweights patients from older data sources and show that this can substantially reduce the impact of these “temporal shifts” on model performance. Methods like this one may ultimately help make AI risk prediction models a more regular and reliable part of clinical care.

## Graphical abstract

**Figure:**
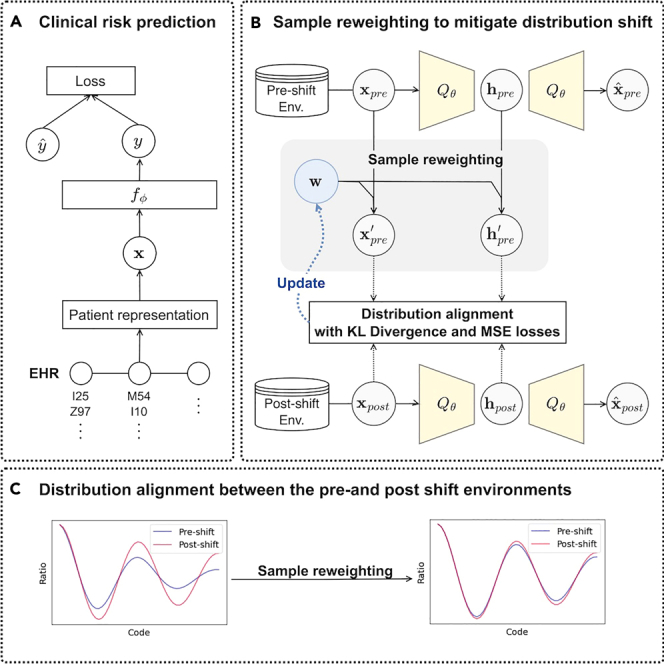

## Teaser

Electronic health records (EHRs) are used in clinical risk prediction to enhance early patient care. Ensuring promising performance, however, generally requires large-scale data for training predictive models. Unfortunately, EHRs are susceptible to temporal data shifts that negatively affect predictive performance, causing challenges in directly leveraging large-scale data. To address this issue, the authors propose a method to mitigate data shifts and their associated impact and experimentally validate that their approach effectively leverages the shifted data, resulting in improved predictive performance.

## Introduction

The availability of large-scale electronic health record (EHR) datasets has led to the development of machine-learning methods for clinical risk prediction that help improve patient care.^1^^,^^2^ Patients’ health records included in EHRs provide useful information for personal health tracking and monitoring^3^^,^^4^^,^^5^^,^^6^ in various tasks in the medical domain.^7^ In this study, we focus on clinical risk prediction, which predicts the risks of future diseases by analyzing previously observed EHR information.

**Figure 1:**
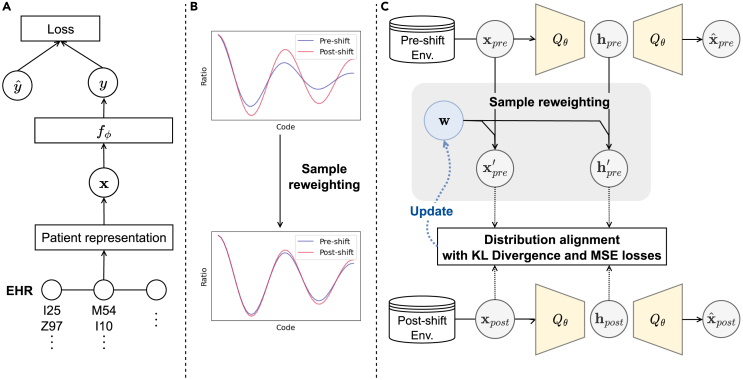
Illustrations of sample reweighting, clinical risk prediction, and the proposed method (A) Diagram of clinical risk prediction. (B) Changes in the distribution of medical codes after sample reweighting to mitigate the distribution shift. (C) Architecture of the proposed method for sample reweighting.

Many deep-learning models have been proposed to predict future diagnoses and have achieved promising results. Choi et al.^8^ developed a recurrent neural-network-based model with reverse time attention modules (RETAIN) to model reverse time-ordered EHR sequences and learn weights for all medical codes, which are used to analyze the codes’ contributions to the prediction. Ma et al.^9^ proposed a bidirectional recurrent neural network (RNN)-based model using different attention mechanisms (Dipole) to model patients’ visits in both time-ordered and reverse time-ordered ways and calculate the weights for previous visits with the attention. Ma et al.^10^ incorporated RNN and multi-head self-attention to consider the personal patient’s health context, extracting interdependencies between clinical features to learn the personal health context. Choi et al.^11^ constructed a graph-based attention model using RNN to model patient visits in the sequential context. Gao et al.^3^ developed a model composed of an RNN and a convolutional module to model disease-stage information for risk prediction. Luo et al.^12^ proposed a time-aware transformer model for health risk prediction. Figure 1A presents a basic diagram of clinical risk prediction using a neural-network-based model. In this diagram, the historical EHRs are fed as input to the model, which then predicts the future diagnosis as an output.

Despite their successes, a fundamental challenge in EHR studies that has not been addressed in previous works is distribution shift. Most machine-learning models are tentatively based on the strong assumption that training and test data points are independently and identically distributed. However, this assumption could be violated for real-world applications, where out-of-distribution (OOD) problems often occur (i.e., the clinical data distribution changes over time). The OOD problems cause significant performance degradation in the testing environment,^13^^,^^14^^,^^15^^,^^16^ which raises serious concern for the application of machine-learning models in the real-world clinical setting.

**Figure 2:**
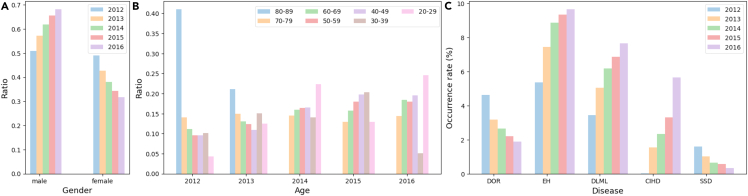
Statistical analysis (A) Gender distribution. (B) Age distribution. (C) Occurrence rates of important diseases that gradually change over time. DOR, dorsalgia; EH, essential (primary) hypertension; DLML, disorders of lipoprotein metabolism and other lipidemias; CIHD, chronic ischemic heart disease; SSD, segmental and somatic dysfunction.

**Figure 3:**
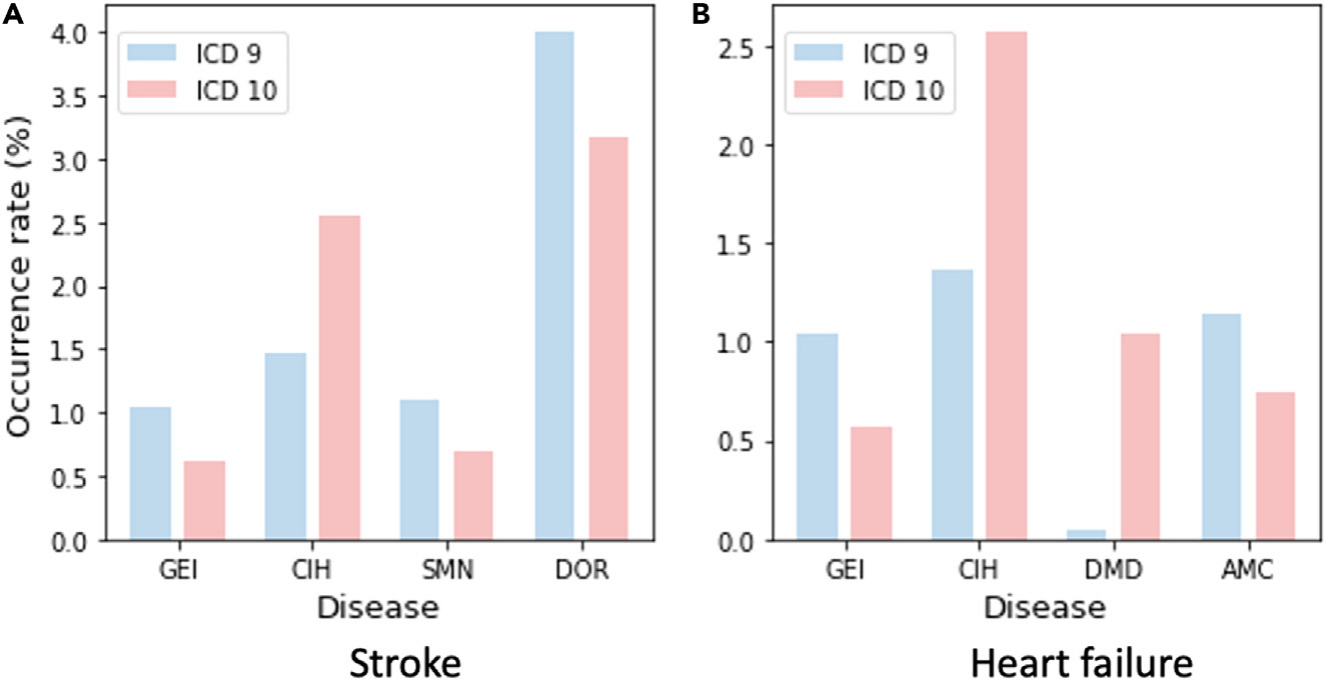
Changes in the occurrence rates of diseases after the transition from ICD-9-CM to ICD-10-CM (A) Changes in important diseases for stroke patients. (B) Changes in important diseases for heart failure patients. CEI, general examination and investigation; CIH, chronic ischemic heart disease; SMN, encounter for screening for malignant neoplasms; DOR, dorsalgia; DMD, dependence on enabling machines and devices; AMC, encounter for other aftercare and medical care.

The distribution shift could appear on EHRs in various ways: (1) difference in the patient population; (2) changes in the practice of medical care; and (3) difference in data formats.^17^ We investigate whether the distribution shift exists in the real-world EHR dataset with respect to the aforementioned ways in Figure 2. Figures 2A and 2B show the distribution of patient demographics (i.e., gender and age). Figure 2C shows that the occurrence rates of some diseases gradually change over time. The accumulation of the changes could cause a critical data shift after several years. Moreover, the transition of the International Classification of Diseases (ICD) codes (e.g., from ICD-9-CM to ICD-10-CM) could also cause data shifts. ICD codes are widely used and play important roles in clinical risk prediction models.^7^^,^^8^^,^^9^ The list of potential diagnosis codes in ICD-10-CM is five times larger than its ICD-9-CM counterpart currently used in practice. When mapping the codes from ICD-9-CM to ICD-10-CM, 27% of the diagnosis codes were convoluted and 3% were found to have no mapping.^18^ Figures 3A and 3B show that the occurrence rates of some diseases change suddenly after the transition from ICD-9 to ICD-10. The frequencies of CEI, CIH, and DMD codes have increased by approximately two times or more since the ICD transition. It is not advisable to apply decision models from previous EHRs that were coded in ICD-9 directly to the latest EHRs without considering the changes in distribution. These changes can result in data shifts and performance decay, leading to inaccurate predictions. Therefore, it is necessary to address the temporal and/or ICD version shifts inherent in EHRs to effectively utilize historical data for predictive models.

Several studies have addressed the OOD problems in medical environments. For instance, Ulmer et al.^19^ investigated uncertainty estimation methods for detecting OOD samples in medical tabular data. However, the study demonstrated that uncertainty estimation methods may not be reliable for OOD detection, since the data are high-dimensional, complex, and noisy. In another work, Luo et al.^6^ proposed a causal representation learning model based on variable decorrelation for diagnosis prediction. This model discovers stable correlations that reflect the causal effect of each feature in different environments, resulting in mitigating bias caused by the distribution shifts between training and inference. Some existing works have focused on data shifts in medical environments. Guo et al.^20^ proposed a domain generalization (DG)^21^-based model that leverages time information as the domain to learn robust and domain-invariant properties across time to mitigate temporal shift. Zhang et al.^22^ proposed AdaDiag, which is based on domain adaption (DA), to handle domain shift. AdaDiag consists of a joint feature extractor that maps input from the source and target domain to the shared feature space, a classifier that performs predictions, and a discriminator for distinguishing the source and target domain.

In this paper, we propose a new method for stable clinical risk prediction to tackle these challenges. We treat the observed EHRs before October 2015 (when the codes are recorded as ICD-9-CM) as pre-shift data and the EHRs observed after October 2015 (when the codes are recorded as ICD-10-CM) as post-shift data. We reweight training patients’ records in pre-shift data to mitigate the distribution shift between the pre- and post-shift data. Figure 1 illustrates the main concepts of the proposed method. Figure 1B presents an example of a distribution shift of medical codes in the post-shift data. After sample reweighting, the distribution changes toward mitigating the distribution shift. Figure 1C shows an architecture of the proposed model for sample reweighting. The proposed model not only directly equalizes the occurrence rate of codes in pre- and post-shift data using mean squared error but also equalizes the probability distribution in the latent space using Kullback-Leibler divergence (KL-divergence).

Note that all the ICD-9-CM codes are mapped to ICD-10-CM codes according to General Equivalence Mappings developed by the Centers for Medicare & Medicaid Services (CMS).^23^ We conduct a comprehensive empirical study on a real-world EHR dataset with different scenarios to demonstrate our hypothesis and to evaluate the effectiveness of our method. To demonstrate our hypothesis that the distribution differences between pre- and post-shift data exist, we first conduct experiments with the following scenarios: (1) we train the existing clinical risk prediction models (e.g., RETAIN, Dipole) for heart failure and stroke risk prediction tasks only with patients in the pre-shift training data, and report the performance on the post-shift test data; (2) we apply our method to the models to evaluate whether our method reduces the distribution shift and improves the performance on the post-shift test data. Experimental results demonstrate our hypothesis and show that our method improves all the baselines.

Our contributions are summarized as follows.

• We investigate the temporal distribution shift on medical codes and the performance differences caused by the shift.
• We design a new method that reweights the pre-shift samples to reduce the distribution shift between the pre- and post-shift samples, learning stable representations for both the pre- and post-shift samples.
• We show that the proposed method not only boosts the prediction performance by sample reweighting but also efficiently leverages the pre-shift historical data through stable learning.
• We conduct a comprehensive experiment to demonstrate our hypothesis and to evaluate the effectiveness of our method.

Experimental results show that our method improves existing predictive models for heart failure and stroke risk, mitigating the distribution shift in diagnosis codes between the pre- and post-shift samples.

## Results

### Data

We conduct our experiments on a real clinical EHR data warehouse, MarketScan Commercial Claims and Encounters (CCAE),^24^ which contains individual-level and de-identified healthcare claims information. MarketScan claims data are primarily used to evaluate health utilization and services. We identify coronary artery disease (CAD) cohorts for which criteria are defined based on ICD codes. There are 1,178,997 patients in total. All patients have a set of medical records including demographic characteristics, time information, drugs, procedures, diagnoses, and other clinically relevant indicators. We consider three categories, namely demographic characteristics, diagnosis, and procedure codes, for study variables. Demographic characteristics consist of age and gender information. Diagnosis codes are defined as ICD codes and consist of 57,089 unique ICD-9/10 codes in MarketScan data.

### Study design

CAD represents a major risk factor for both heart failure^25^^,^^26^ and stroke.^27^^,^^28^ In this work, we focus on clinical risk prediction of whether a patient will suffer heart failure or stroke in the future. The definitions of heart failure and stroke are presented in Tables S1 and S2. We conduct a case-control study, a type of epidemiological observational study, on clinical risk prediction tasks. The case-control study identifies two groups of subjects with different diseases but similar conditions and compares them to discover factors that contribute to the differences. Patients diagnosed with heart failure or stroke are collected as case patients. Then, for each case patient, a control patient with the same demographics and characteristics, such as the same age, gender, and number of visits, is selected.

**Figure 4:**
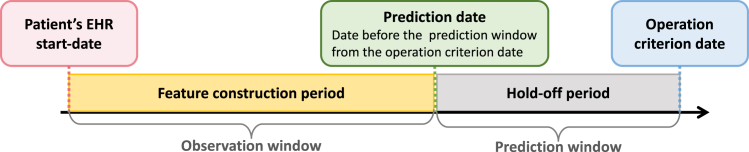
Settings to construct the experimental EHR data for clinical risk prediction tasks The operation criterion date refers to the date of the EHR diagnosed with target diseases (case patients) or the end date of the EHR (control patients). The prediction date represents the date before the prediction window, tracking from the operation criterion date.

To predict the diagnosis of heart failure or stroke at some future time, it is necessary to set operation criterion and prediction dates. Figure 4 shows the settings to construct the experimental EHR data from the large database for early prediction tasks. The operation criterion date indicates the date of the future diagnosis to be predicted. The prediction date refers to the date before the prediction window from the operation criterion date to make a prediction for future diagnosis. Each patient’s EHR data are then split into an observation window and a prediction window. The prediction window includes the medical records for the last 360, 180, or 90 days tracing back from the operation criterion date. The observation window contains all the records before the prediction window and is used for analysis. For example, if a patient is diagnosed with heart failure on October 5, 2014, the records up to October 1, 2013 are included in the observation window for predicting heart failure with a prediction window of 360 days. In the case of the case patients, the date of the EHR diagnosed with heart failure or stroke is set as the operation criterion date. In the case of the control patients, the last date of the EHR is set as the operation criterion date. When selecting control patients for the case-control study, the prediction date is also included in the characteristics similar to those of the case patients to accurately analyze EHR data over time. In addition, to ensure that there are sufficient medical events to predict the future diagnosis, only patients with more than ten records (visits) in the observation window are selected for analysis.

#### Data pre-processing

We pre-process the EHR data by chronologically concatenating the medical records for each patient according to previous works,^7^^,^^29^ as the temporal information is critical. Thus, all patients are represented as a variable-length sequence of records equal to the corresponding number of visits. For convenience, all patients’ records are padded to the same size based on the maximum number of visits, and the padding records are not medically meaningful. For equivalence between codes of ICD-9-CM and ICD-10-CM versions, all medical codes in the dataset originally coded as ICD-9-CM are pre-converted into ICD-10-CM’s codes before the experiments according to General Equivalence Mappings developed by CMS.^23^ In our study we only consider the first three letters, which are representative categories including more detailed codes, to reduce the number of diagnosis codes. To address the potential loss of information resulting from reducing ICD codes to a low number of letters, we conducted a validation process to ensure that the codes retained sufficient granularity to capture meaningful differences between patients’ diagnoses. Specifically, we compared the performances of models trained with full-length ICD codes and shortened codes, ranging from 5-letter to 1-letter codes. Our results show that using the full-length codes led to a lower area under the receiver-operating characteristics curve (AUROC) compared to the shortened codes. The results can be attributed to the lower frequency of 5-letter codes, which may pose challenges in effectively learning their embeddings. Conversely, using shortened codes did not adversely impact the model’s performance. For the heart failure prediction problem with a 360-day prediction window, the number of unique codes is 6,629 for full-length codes and 1,474 for three-letter codes. We found that using the first three or two letters of the ICD codes resulted in optimal performance. However, since 3-letter codes include category information about the disease codes, we decided to use only the first three letters of the ICD codes in our study. The results of the experiment can be found in Table S3.

#### Data shift

**Figure 5:**
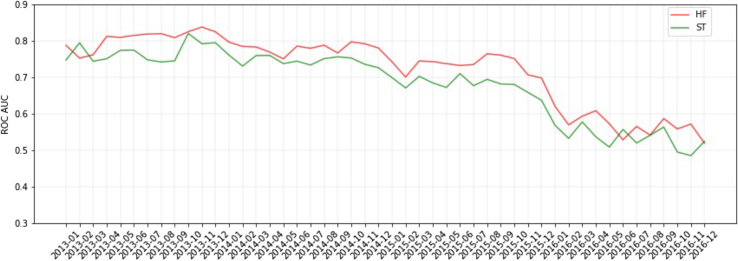
Visualization of performance per month for heart failure and stroke risk prediction The x axis indicates the months and the y axis represents AUROC scores. The model is trained only with patients up to 2013. HF, heart failure; ST, stroke,

We observe that the occurrence rates of some important diseases gradually change over time and also change suddenly after the transition from ICD-9 to ICD-10 in Figures 2 and 3. These changes could cause distribution shifts and severe performance decay. To demonstrate the existence of the distribution shift in EHRs and how it affects the model performance, we report the prediction performance trend over time with a neural-network-based model that is trained and optimized only for patients whose prediction date is up to December 31, 2013. Figure 5 shows the prediction performance per month based on the prediction date for heart failure and stroke risk prediction tasks. The predictive model is trained only with patients whose prediction date is up to 2013. The x axis indicates the months and the y axis represents AUROC scores. As illustrated on the graph, the score gradually decreases over time, with a rapid decline observed from October to December 2015. This finding indicates that there is a significant distribution shift before and after October 2015, highlighting the need to address temporal shifts when working with EHRs. To further investigate the potential influence of gender distribution on clinical risk prediction, we also compare the average AUROC scores for the overall population, males, and females by year. Figure S1 shows the results for the model trained with patients up to 2013. Our analysis reveals that there is no significant difference in performance based on gender. As a result, we focus on the data shift rather than the gender distribution.

#### Experimental setting

**Figure 6:**
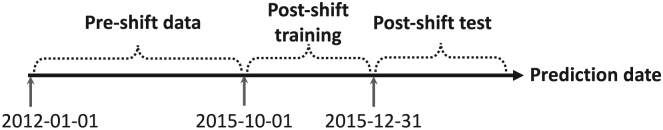
Experimental settings for the data shift We use the EHRs with the prediction date prior to 2015-10-01 as the pre-shift data. The EHRs with the prediction date from 2015-10-01 to 2015-12-31 are used as the post-shift training data to reweight the pre-shift data, and EHRs with the prediction date after 2016-01-01 are used as the post-shift test data to evaluate the prediction performance.

Based on our findings, we treat EHRs before and after October 2015 as pre-shift and post-shift data, respectively. Aiming to decrease the significant performance difference between the pre-shift and post-shift data, we design our model with the following settings. Figure 6 shows the experimental settings of clinical risk prediction tasks for our model. The EHRs with the prediction date prior to October 1, 2015 are used as the pre-shift data. The pre-shift data are further split into the pre-shift training, validation, and test data to train, optimize, and evaluate the predictive model, respectively. To mitigate the distribution shift between the pre-shift and post-shift data, the post-shift data with the prediction date from October 1, 2015 to December 31, 2015 are used as the post-shift training data to reweight the pre-shift training data. The post-shift data with the prediction date after January 1, 2016 are then used as the post-shift test data to evaluate the prediction performance. The statistics of the dataset for heart failure and stroke risk prediction tasks are described in Tables 1 and 2.

**Table 1:** Statistics of the dataset for heart failure prediction

Prediction window	360 days					180 days					90 days				
Data	Pre-shift			Post-shift		Pre-shift			Post-shift		Pre-shift			Post-shift	
	Train	Valid	Test	Train	Test	Train	Valid	Test	Train	Test	Train	Valid	Test	Train	Test
No. of unique codes	1,474					1,490					1,521				
No. of patients	26,408	8,940	8,926	1,706	3,418	30,290	10,200	10,178	2,616	7,408	28,926	9,740	9,750	3,024	9,798
No. of visits	524,898	177,628	175,674	34,544	67,732	544,648	184,014	185,064	51,624	147,744	524,262	176,252	175,692	59,002	194,122
Avg. no. of visits per patient	18	18	18	20	19	18	18	18	19	19	18	18	18	19	19
Avg. no. of codes per visit	2.25	2.26	2.24	2.43	2.53	2.31	2.32	2.30	2.42	2.53	2.34	2.36	2.34	2.42	2.52
Max. no. of codes per visit	28	34	25	16	19	31	34	22	15	29	28	20	34	27	29

**Table 2:** Statistics of the dataset for stroke prediction

Prediction window	360 days					180 days					90 days				
Data	Pre-shift			Post-shift		Pre-shift			Post-shift		Pre-shift			Post-shift	
	Train	Valid	Test	Train	Test	Train	Valid	Test	Train	Test	Train	Valid	Test	Train	Test
No. of unique codes	1,472					1,476					1,500				
No. of patients	24,738	8,278	8,314	1,380	3,234	26,408	8,940	8,926	2,100	6,346	24,866	8,348	8,342	2,394	8,248
No. of visits	458,674	152,372	153,298	27,674	64,018	483,676	162,976	163,564	41,572	126,478	455,476	153,700	152,200	47,378	164,056
Avg. no. of visits per patient	18	18	18	20	19	18	18	18	19	19	18	18	18	19	19
Avg. no. of codes per visit	2.26	2.25	2.25	2.42	2.46	2.31	2.31	2.31	2.41	2.51	2.34	2.34	2.35	2.43	2.53
Max. no. of codes per visit	38	25	28	30	44	34	32	38	30	21	34	38	28	30	21

We compare the prediction performances of models trained with the original pre-shift training data and the reweighted pre-shift training data, respectively. We apply our method to existing clinical risk prediction models. Our method reweights the pre-shift patients’ EHRs to make their distributions similar to that of the post-shift patients, mitigating the distribution shift between them for stable learning. Moreover, we adopt KL loss to learn stable and similar patient representation extracted from the pre-shift and post-shift data. In Tables 3, 4, and 5, Basic and Weighted represent the results of the existing methods and the proposed method, respectively. Accuracy, area under the precision-recall curve (AUPRC), and AUROC are used as performance measurements.

**Table 3:** Comparison of prediction performance on the post-shift test set for heart failure prediction

Prediction window		360 days		180 days		90 days	
		AUPRC	Accuracy	AUPRC	Accuracy	AUPRC	Accuracy
LSTM	Basic	0.5730 ± 0.017	0.5301 ± 0.002	0.6677 ± 0.005	0.5840 ± 0.006	0.7018 ± 0.006	0.6247 ± 0.007
	Weighted	0.5865 ± 0.015	0.5319 ± 0.002	0.6763 ± 0.007	0.5859 ± 0.007	0.7133 ± 0.009	0.6344 ± 0.008
GRU	Basic	0.5781 ± 0.005	0.5309 ± 0.002	0.6718 ± 0.004	0.5889 ± 0.005	0.7095 ± 0.003	0.6309 ± 0.004
	Weighted	0.5964 ± 0.006	0.5336 ± 0.003	0.6803 ± 0.004	0.5912 ± 0.004	0.7144 ± 0.005	0.6348 ± 0.005
Dipole	Basic	0.5905 ± 0.002	0.5322 ± 0.002	0.6757 ± 0.002	0.5937 ± 0.004	0.7095 ± 0.002	0.6308 ± 0.002
	Weighted	0.5968 ± 0.003	0.5330 ± 0.002	0.6781 ± 0.003	0.5977 ± 0.005	0.7171 ± 0.002	0.6375 ± 0.003
RETAIN	Basic	0.5934 ± 0.006	0.5414 ± 0.003	0.6726 ± 0.002	0.5912 ± 0.004	0.7128 ± 0.003	0.6362 ± 0.003
	Weighted	0.5971 ± 0.006	0.5428 ± 0.003	0.6763 ± 0.003	0.5983 ± 0.004	0.7156 ± 0.004	0.6422 ± 0.003
ConCare	Basic	0.5946 ± 0.004	0.5421 ± 0.001	0.6756 ± 0.002	0.5866 ± 0.002	0.7123 ± 0.003	0.6353 ± 0.003
	Weighted	0.5965 ± 0.005	0.5491 ± 0.001	0.6781 ± 0.002	0.5906 ± 0.003	0.7140 ± 0.003	0.6437 ± 0.003
StageNet	Basic	0.5911 ± 0.003	0.5305 ± 0.001	0.6743 ± 0.003	0.5829 ± 0.002	0.7057 ± 0.002	0.6326 ± 0.001
	Weighted	0.5946 ± 0.004	0.5441 ± 0.002	0.6787 ± 0.005	0.5899 ± 0.004	0.7148 ± 0.002	0.6410 ± 0.002

The baseline and proposed method are denoted by Basic and Weighted, respectively. The average score and standard deviation under ten trials are reported. The results for other metrics can be found in Table S4.

**Table 4:** Comparison of prediction performance on the post-shift test set for stroke prediction

Prediction window		360 days		180 days		90 days	
		AUPRC	Accuracy	AUPRC	Accuracy	AUPRC	Accuracy
LSTM	Basic	0.5610 ± 0.011	0.5212 ± 0.003	0.5972 ± 0.008	0.5522 ± 0.002	0.6340 ± 0.006	0.5685 ± 0.008
	Weighted	0.5801 ± 0.014	0.5253 ± 0.004	0.6145 ± 0.011	0.5573 ± 0.003	0.6441 ± 0.009	0.5792 ± 0.009
GRU	Basic	0.5666 ± 0.006	0.5210 ± 0.002	0.6136 ± 0.004	0.5574 ± 0.006	0.6452 ± 0.006	0.5815 ± 0.006
	Weighted	0.5746 ± 0.008	0.5278 ± 0.003	0.6294 ± 0.005	0.5608 ± 0.007	0.6492 ± 0.008	0.5843 ± 0.006
Dipole	Basic	0.5702 ± 0.003	0.5275 ± 0.002	0.6157 ± 0.003	0.5592 ± 0.003	0.6460 ± 0.003	0.5827 ± 0.003
	Weighted	0.5900 ± 0.005	0.5290 ± 0.003	0.6260 ± 0.005	0.5601 ± 0.003	0.6528 ± 0.006	0.5920 ± 0.004
RETAIN	Basic	0.5756 ± 0.003	0.5259 ± 0.003	0.6222 ± 0.003	0.5563 ± 0.004	0.6382 ± 0.005	0.5781 ± 0.003
	Weighted	0.5869 ± 0.004	0.5279 ± 0.002	0.6339 ± 0.005	0.5598 ± 0.005	0.6519 ± 0.007	0.5986 ± 0.003
ConCare	Basic	0.5762 ± 0.006	0.5261 ± 0.005	0.6261 ± 0.002	0.5606 ± 0.003	0.6464 ± 0.004	0.5852 ± 0.002
	Weighted	0.5862 ± 0.008	0.5343 ± 0.005	0.6356 ± 0.004	0.5669 ± 0.003	0.6517 ± 0.007	0.5872 ± 0.003
StageNet	Basic	0.5684 ± 0.006	0.5201 ± 0.001	0.6263 ± 0.005	0.5594 ± 0.004	0.6419 ± 0.004	0.5780 ± 0.002
	Weighted	0.5776 ± 0.007	0.5216 ± 0.002	0.6323 ± 0.006	0.5606 ± 0.005	0.6511 ± 0.007	0.5849 ± 0.003

The baseline and proposed method are denoted by Basic and Weighted, respectively. The average score and standard deviation under ten trials are reported. The results for other metrics can be found in Table S5.

**Table 5:** Comparison of prediction performance on the post-shift test set for heart failure and stroke prediction

Prediction window		360 days		180 days		90 days	
		AUPRC	Accuracy	AUPRC	Accuracy	AUPRC	Accuracy
HF	Basic	0.5905 ± 0.002	0.5322 ± 0.002	0.6757 ± 0.002	0.5937 ± 0.004	0.7095 ± 0.002	0.6308 ± 0.002
	AdaDiag	0.5896 ± 0.028	0.5296 ± 0.011	0.6760 ± 0.013	0.5935 ± 0.002	0.7104 ± 0.007	0.6319 ± 0.002
	DG	0.5906 ± 0.009	0.5323 ± 0.005	0.6769 ± 0.003	0.5962 ± 0.005	0.7127 ± 0.002	0.6282 ± 0.001
	Weighted	0.5968 ± 0.003	0.5330 ± 0.002	0.6781 ± 0.003	0.5977 ± 0.005	0.7171 ± 0.002	0.6375 ± 0.003
ST	Basic	0.5702 ± 0.003	0.5275 ± 0.002	0.6157 ± 0.003	0.5592 ± 0.003	0.6460 ± 0.003	0.5827 ± 0.003
	AdaDiag	0.5697 ± 0.009	0.5290 ± 0.003	0.6180 ± 0.014	0.5594 ± 0.002	0.6472 ± 0.011	0.5830 ± 0.003
	DG	0.5726 ± 0.007	0.5283 ± 0.003	0.6254 ± 0.002	0.5603 ± 0.001	0.6503 ± 0.003	0.5832 ± 0.001
	Weighted	0.5900 ± 0.005	0.5290 ± 0.003	0.6260 ± 0.005	0.5601 ± 0.003	0.6528 ± 0.006	0.5920 ± 0.004

Basic, AdaDiag, and DG are baseline methods, and Weighted refers to the proposed method. We use the Dipole as a backbone network for both DG and Weighted. The average score and standard deviation under ten trials are reported. Results of statistical tests can be found in Table S6.

### Results for clinical risk prediction

Tables 3 and 4 show the performances of clinical risk prediction on the post-shift test set as measured by AUPRC and accuracy scores for heart failure and stroke, respectively. The proposed method (marked as weighted in the tables) improves all baselines (marked as basic) on both AUPRC and accuracy scores. The results demonstrate that the proposed method mitigates the distribution shift and thus provides more robust performance for new patients that differ from training patients. Such findings indicate the advantage of the proposed method to learn stable representations for the post-shift data by sample reweighting. When comparing the performance of baseline models, the advanced models generally exhibit better overall performance than the simpler models such as GRU and LSTM. Specifically, ConCare and StageNet achieve superior performance across the board. The results of the experiment on other metrics, including AUROC, precision, and recall, can be found in Tables S4 and S5.

We also compare the performance of the proposed method with DG and AdaDiag methods, which are existing tools to alleviate temporal data shifts. For a fair comparison, both DG and AdaDiag methods utilize the post-shift training data for model training. DG and the proposed method (weighted) employ the Dipole model as the backbone network. Table 5 shows the performance results on AUPRC and accuracy. While all the comparative models outperform the basic model that does not utilize the post-shift training data in most cases, the proposed method exhibits the highest improvement in almost all cases. This demonstrates that the proposed method effectively mitigates data distribution shifts through the sample reweighting approach. To assess the statistical significance of the differences between the performances of the proposed method and existing works, we conduct Friedman and Wilcoxon tests on AUPRC scores. We apply the Friedman test with the null hypothesis ($H_{0}$) that there is no statistically significant difference between the performances of the methods, while the alternative hypothesis ($H_{1}$) assumes the presence of the difference. In addition, the Wilcoxon test is applied to test the null hypothesis $H_{0}$ that there is no statistically significant difference between the performances of the top two methods, weighted and DG, and the alternative hypothesis $H_{1}$ that there is a significant difference. Table S6 presents the results of both tests, including the p values obtained from ten repeated experiments. Based on the results, we rejected the null hypothesis at a significance level of α=0.05, indicating statistically significant differences among the performances of the methods.

#### The usefulness of the proposed method

We observe the temporal distribution shift in EHR records as the prediction performance changes over time. In particular, the performance decreases significantly as of October 2015, so we present our method to mitigate the distribution shift based on that time. Although we have demonstrated the effectiveness of our method through previous experiments, we further conduct an additional experiment to prove the usefulness of the proposed method. The settings for the additional experiment are as follows. (1) We randomly split the post-shift data (EHRs after October 2015) into the training, validation, and test data, then train the model only with the training data. The prediction performance is reported on the post-shift test data. (2) We further train the model with the pre-shift data reweighted by the proposed method using the post-shift training data. The prediction performance is also reported on the post-shift test data. As shown in Table 6, the experimental results using the weighted pre-shift data (denoted as pre-shift training) achieve higher performance compared to only using the post-shift training data (denoted as post-shift training) by about 17.2% on AUROC. This experiment shows that our method not only efficiently leverages large amounts of historical pre-shift data for model training but also improves performance.

**Table 6:** Comparison of prediction performances on AUROC and accuracy using the post-shift data and both pre- and post-shift data as training sets

Prediction window		360 days		180 days		90 days	
		AUROC	Accuracy	AUROC	Accuracy	AUROC	Accuracy
HF	Post-shift training	0.5821 ± 0.013	0.5399 ± 0.015	0.5795 ± 0.009	0.5593 ± 0.010	0.6182 ± 0.015	0.5782 ± 0.020
	Pre-shift training	0.6597 ± 0.006	0.6062 ± 0.008	0.7029 ± 0.004	0.6490 ± 0.008	0.7282 ± 0.003	0.6630 ± 0.006
ST	Post-shift training	0.5325 ± 0.020	0.5059 ± 0.006	0.5357 ± 0.022	0.5149 ± 0.022	0.5661 ± 0.012	0.5200 ± 0.020
	Pre-shift training	0.6088 ± 0.008	0.5642 ± 0.014	0.6317 ± 0.007	0.5960 ± 0.008	0.6716 ± 0.005	0.6255 ± 0.004

The average score and standard deviation under ten trials are reported. Note that we have access to small-scale post-shift data (i.e., 3 months records) in the post-shift training setting and large-scale pre-shift data (i.e., more than 3 years) in the pre-shift training setting. We use the GRU model in the two settings.

#### Distribution shift

**Figure 7:**
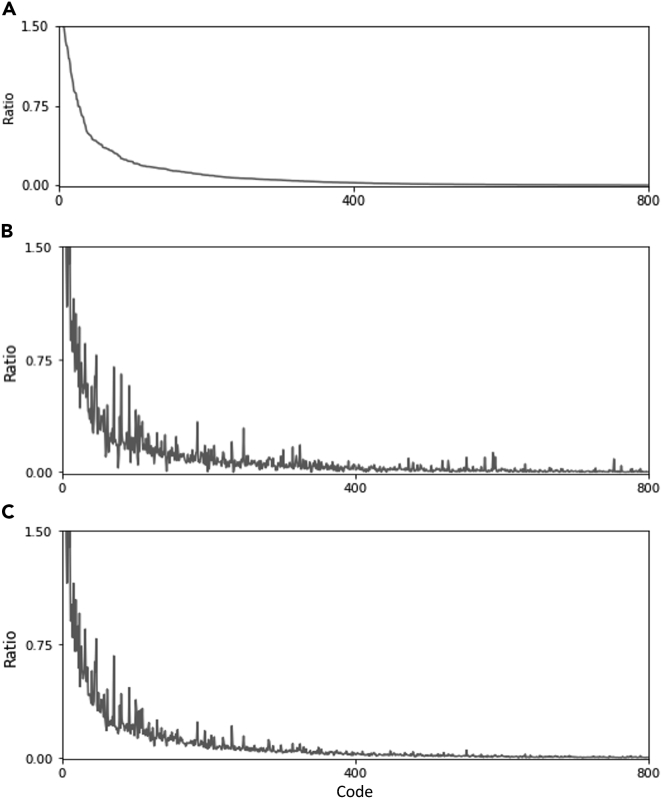
Visualization of code distribution The x and y axes indicate the codes and ratios, respectively. x is set in descending order of the ratios on the pre-shift training data. (A) Distribution of the pre-shift training data. (B and C) Post-shift test data (B) and the reweighted pre-shift training data (C).

The proposed method mitigates the distribution shift in EHRs, especially in the medical codes. Figure 7A–Cshow the code distributions for the pre-shift training set, post-shift test set, and reweighted training set, respectively. Here, the x and y axes indicate the codes and ratios of them, respectively. The x axis is set in descending order of the ratios on the pre-shift training data. As shown in Figures 7A and 7B, there exists a distribution shift between the pre-shift training and post-shift tests. Noticeably, the distribution of the reweighted training set (i.e., Figure 7C) becomes very similar to the post-shift test set, compared to Figure 7A. This result also evaluates that the sample weighting mitigates the distribution shift.

#### Ablation study

We conduct an ablation study to investigate whether each component of our model actually contributes to the predictive performance. Starting from the original version of the proposed model, each component is independently excluded to construct some model variants, proposed method without $L_{mse}$ and proposed method without $L_{KL}$. Table 7 shows the results of the ablation study. The prediction performance is reduced when each component is removed. These results demonstrate the effectiveness of directly equalizing the distributions of the codes and reducing the difference between the latent distributions in the sequential context.

**Table 7:** Ablation study for the proposed method

Model	AUROC
Proposed method	0.6185
Proposed method without $L_{mse}$	0.6057
Proposed method without $L_{KL}$	0.6031

The model is based on GRU, and the prediction period is 360 days.

## Discussion

### Principal results

In this study, we investigate the temporal distribution shift in diagnosis codes and the performance degradation that accompany the shift. Prediction performance tends to decrease slightly over time but decreases significantly since October 2015 when the ICD version was changed from ICD-9-CM to ICD-10-CM. We investigate that the post-shift data (EHRs after October 2015) achieves significantly lower performance for a predictive model trained on the pre-shift data (EHRs before October 2015), due to the distribution shift. Conversely, even if it is trained with the post-shift data, it also provides poor performance due to the small number of data. This suggests that the model trained with the past EHRs coded as ICD-9-CM cannot be generalized to the EHRs coded as ICD-10-CM and thus be exploited at all.

In this work, we address the challenges of the performance degradation over time and the ICD version changes by stable learning, which learns stable representation for both pre- and post-shift data, mitigating the distributional shift between them. Experiments on the real-world dataset demonstrate that our method not only improves state-of-the-art models but also generalizes prediction performance for new patients that differ from training patients. Our experimental findings are significant because it creates new chances for EHR studies. The experimental results showing that the past EHRs improve prediction performance provide many research opportunities to explore and pursue the benefits of the past EHRs. Furthermore, our method builds a bridge between different datasets, providing generalized performance and thus allowing the data to be cross-used.

### Conclusion

Clinical risk prediction is crucial for improving healthcare quality. We investigate that there exist inconsistencies in the distributions of the diagnosis codes depending on time and ICD versions, resulting in the distribution shift between them. In this paper, we propose a novel method to address these issues for clinical risk prediction, learning the sample weights in pre-shift data to mitigate the distribution shift between the pre- and post-shift data. The proposed method not only directly equalizes the occurrence rate of codes in pre- and post-shift data but also equalizes the probability distribution in latent space using KL-divergence. The experimental results demonstrate that our proposed method degrades the distribution shift and thus improves the prediction performance.

## Experimental procedures

### Resource availability

#### Lead contact

Further information and requests for resources and reagents should be directed to and will be fulfilled by the lead contact, Ping Zhang (zhang.10631@osu.edu).

#### Materials availability

This study did not generate any new materials.

### Clinical risk prediction definitions and basic notations

We use uppercase and bold letters (e.g., **X**) for matrices, lowercase and bold letters (e.g., **x**) for vectors, and lowercase letters (e.g., *x*) for scalars. Table 8 summarizes the notations used in our method.

**Table 8:** Notation definitions

Notation	Description
$D_{pre}\equiv {\left\{X_{i},{\hat{y}}_{i}\right\}}_{i=1}^{\left|D_{pre}\right|}$	pre-shift training data
$D_{post}\equiv {\left\{X_{i},{\hat{y}}_{i}\right\}}_{i=1}^{\left|D_{post}\right|}$	post-shift training data
$X_{i}$	*i*th patient’s EHR sequence
$x_{i,t}$	*i*th patient’s *t*th EHR
w	sample weights
${\hat{y}}_{i}$	label for $X_{i}$
$y_{i}$	prediction for $X_{i}$
$d^{pre},d^{post}$	code distributions for $X^{pre},X^{post}$
$h^{pre},h^{post}$	latent distributions for $X^{pre},X^{post}$
$Z_{i}$	latent representation for $X_{i}$
α,β	weights to control losses
*Q*	encoder network
*P*	decoder network
*F*	classifier

#### EHR sequence

The EHR data for each patient are represented as a sequence of visits in the order of their occurrence. Each visit of the sequence has a set of varying numbers of diagnosis codes. Thus the *v*th visit of the *i*th patient is expressed as a binary vector $x_{i,v}\in {\left\{0,1\right\}}^{C}$, where *C* is the number of unique diagnosis codes, and a value of 1 for the *k*th coordinate (i.e., $x_{i,v,k}=1$) indicates that the *k*th code is recorded at the *v*th visit of the *i*th patient. The EHR sequence for the *i*th patient is denoted by $X_{i}=\left[x_{i,1},x_{i,2},\cdots ,x_{i,t_{i}}\right]$ where $t_{i}$ is the number of visits for the *i*th patient.

#### Clinical risk prediction

Given the EHR sequence $X_{i}=\left[x_{i,1},x_{i,2},\cdots ,x_{i,T}\right]$, the goal of health risk predictive modeling in this paper is to predict the target disease at the end of the sequence. The label for the *i*th patient is denoted by ${\hat{y}}_{i}\in \left\{0,1\right\}$, because we focus on two tasks to predict heart failure and stroke disease separately.

### Architecture

The proposed framework consists of two steps: (1) sample reweighting that learns the sample weights for the pre-shift training patients using the corresponding EHR sequences to mitigate the temporal distribution shift between the pre- and post-shift training data; (2) classification that learns stable representations from the EHR sequences with the sample weights to predict the best future diagnosis. Figure 1C shows the architecture of the proposed method for sample reweighting.

#### Sample reweighting

We propose to learn sample weights for the pre-shift training samples to mitigate the distribution shift on diagnosis codes between the pre- and post-shift training sets. We use two approaches; directly equalize the occurrence rates of codes in the pre- and post-shift training samples and equalize the probability distribution of them in latent space.

To directly equalize the distributions of the codes, we first compute the target distribution of the codes for the post-shift samples by Equations 1 and 2:

(Equation 1) $$s_{k}^{post}=\sum_{x\in D_{post}}\sum_{j=1}^{T}x_{,j,k}\text{,}$$

(Equation 2) $$d_{k}^{post}=\frac{s_{k}^{post}}{\sum_{k=1}^{\left|C\right|}s_{k}^{post}}\text{,}$$

where $D_{post}$ is the post-shift training data and *T* is the number of visits for the corresponding patient. We use $w\in R_{+}^{\left|D_{pre}\right|}$ to denote the sample weights, where $D_{pre}$ is the pre-shift training data. The code distribution $d^{pre}$ for $D_{pre}$ can be obtained by Equations 3 and 2.

(Equation 3) $$s_{k}^{pre}=\sum_{i=1}^{\left|D_{pre}\right|}\sum_{j=1}^{t}w_{i}\cdot x_{i,j,k}\text{.}$$

The difference between the pre- and post-shift training distributions is then computed using mean squared error (MSE). The loss is as follows:

$$L_{mse}=\frac{1}{C-1}\sum_{k=1}^{C}{\left(d_{k}^{pre}-d_{k}^{post}\right)}^{2}\text{.}$$

The MSE loss directly adjusts the occurrence rate of the diagnosis codes and thus mitigates the distribution differences between training and test sets, but it ignores the sequential context of EHRs. That is, the relation between a patient’s visits is not considered.

To address this issue and further force the distributions to be similar, we map the samples to latent representations via an auto-encoder network.^30^ The main idea is to construct an embedding space from which the abstract information of the sequence for all visits is generated and to learn robust weights in the latent space. After embedding, the latent features for the training samples are weighted. We then minimize Kullback-Leibler divergence (KL-divergence) between two distributions in the latent space.

We first map pre- and post-shift training samples to the sequence of latent representations, **z**, with the auto-encoder model whose encoder network is $Q:R^{T\times \left|C\right|}\to R^{T\times F}$ and decoder network is $P:R^{T\times F}\to R^{T\times \left|C\right|}$. Here *T* and *F* are the number of visits and the dimension of latent features from *Q*, respectively. The auto-encoder model is first trained with both pre- and post-shift data before training the sample weights to learn useful latent representations of the input code space. The reconstruction loss is as follows:

(Equation 4) $$\begin{array}{l} {\hat{x}}_{i}=P\left(Q\left(x_{i}\right)\right)\\ L_{reconst}=\sum_{x\in D_{pre},D_{post}}{\left(x_{i}-{\hat{x}}_{i}\right)}^{2} \end{array}\text{.}$$

After training the auto-encoder mode with Equation 4, the sequence of latent representations for *i*th patient is obtained as follows:

(Equation 5) $$Z_{i}=\left[z_{i,1},z_{i,2},\cdots ,z_{i,T}\right]=\left[Q\left(x_{i,1}\right),Q\left(x_{i,2}\right),\cdots ,Q\left(x_{i,T}\right)\right]\text{,}$$

$where\;Z_{i}$ reflects the sequence of diagnosis codes for all visits in the order of their occurrence. The pre- and post-shift training distributions in the latent space are then computed as

(Equation 6) $$h^{pre}=\frac{1}{\left|D_{pre}\right|}\sum_{i=1}^{\left|D_{pre}\right|}w_{i}\cdot Z_{i}\text{,}$$

(Equation 7) $$h^{post}=\frac{1}{\left|D_{post}\right|}\sum_{i=1}^{\left|D_{post}\right|}Z_{i}\text{.}$$

The KL loss between two latent distributions is expressed in Equation 8:

(Equation 8) $$L_{KL}=h^{post}\cdot \log\frac{h^{post}}{h^{pre}}\text{.}$$

We iteratively optimize sample weights by (Equation 9), (Equation 10). Here *α* and *β* are the coefficients that control MSE and KL-divergence constraints, respectively, and $\Delta =\left\{w\in R_{+}^{n}\right\}$. We consistently consider non-negative weights. Positive weights represent the relative importance of samples, enabling the model to effectively learn from significant samples. Conversely, the use of negative weights may result in the model considering samples in the opposite manner, which could lead to confusion and misinterpretation of the intended meaning of the weights. w is also regularized so that the sum of w equals the number of data. The reason for this regularization is that if the sample weights are too small or large, it can cause instability or non-convergence of the model during training. By constraining the sum of sample weights, the model training can be stabilized and facilitated to converge, thereby enhancing the performance and robustness of the model:

(Equation 9) $$L_{w}=\alpha \cdot L_{MSE}+\beta \cdot L_{KL}+{\left(\sum_{i=1}^{N}w_{i}-N\right)}^{2}\text{,}$$

(Equation 10) $$w^{t+1}=\arg\min_{w\in \Delta }L_{w}\text{.}$$

#### Classification

The clinical risk prediction is conducted with a classification network $f:R^{T\times \left|C\right|}\to R$. Given the trained sample weights, the weights are fixed and then multiplied by the classification losses for the corresponding training data to train the classification model. Samples with smaller weights have less impact on the model training, and larger weights have more impact. The weighted losses allow learning stable representations for both the pre- and post-shift training data.

Our algorithm iteratively optimizes the prediction function *f* as follows:

(Equation 11) $$f^{t+1}=\arg\min_{f}\sum_{X\in D_{pre}}w_{i}\cdot L_{label}\left(f\left(X,{\hat{y}}_{i}\right)\right)\text{,}$$

where $L_{label}\left(\cdot \right)$ represents the binary cross-entropy loss function.

In the training phase, we optimize the predictive model parameters with the weighted training samples. On the other hand, in the inference phase, the model directly predicts the label without any sample weights.

#### Optimization

To apply the proposed method, we use a two-stage optimization process as follows. First the sample weights w are trained by minimizing $L_{w}$ on the pre- and post-shift training data, $D_{pre}$ and $D_{post}$. The trained weights w are then used in the training of the classification network *f* in which the classification losses for $D_{pre}$ are multiplied by the corresponding weights. The loss $L_{label}$ is minimized for prediction.

### Baseline methods

We apply our method to several deep-learning-based models for health risk prediction to validate the effectiveness of our method. All models only use historical diagnoses as input without additional information such as ontology and temporal intervals for a fair comparison. The baseline models we use are described as follows. LSTM^31^: the variant of RNN with a long-short term gating mechanism. GRU^32^: the variant of RNN. Dipole^9^: the bidirectional recurrent-neural-network-based model with attention mechanisms. Dipole models patients’ visits in both time-ordered and reverse time-ordered ways and calculates the weights for previous visits with attention. RETAIN^8^: the RNN-based model with reverse time attention modules to model reverse time-ordered EHR. The attention learns weights for all medical codes, which are used to analyze the codes’ contributions to the prediction. ConCare^10^: the RNN-based model with multi-head self-attention to consider the personal patient’s health context. ConCare extracts interdependencies between clinical features to learn the personal health context. StageNet^3^: The neural-network-based model with an LSTM module and a convolutional module to model disease-stage information for risk prediction.

To further evaluate our method, we compare our method with existing methods for mitigating temporal data shift. DG refers to a DG-based model that learns robust representation over time.^20^ DG leverages the aforementioned baseline model as its backbone network and has a one-layer adversarial network after the last hidden layer. Each year is set in a different domain, and both pre- and post-shift training sets are utilized for the model training phase. AdaDiag is a DA-based model that consists of a transformer encoder, domain discriminator, and disease classifier. The pre- and post-shift training sets are set to the source and target domains, respectively.

### Implementation and evaluation

All models are implemented by PyTorch.^33^ We use the ADAM algorithm on a mini-batch of 32 patients to optimize the predictive model. The optimal hyper-parameters are found with the validation data in the training phase. The training phase stops when the validation metric is not improved for ten epochs, then test performance is reported. Hyper-parameters used by all baseline methods include the learning rate, the number of hidden nodes, and the number of hidden layers. The ranges of the hyper-parameters are {1e−3, 1e−4} for the learning rate, {128, 256, 512} for the number of hidden nodes, and {2, 3} for the number of layers. For the proposed method, the hyper-parameters used to optimize the auto-encoder include the number of hidden nodes. The learning rate and the number of epochs for training the auto-encoder are fixed at 0.001 and 1,000, respectively. Additionally, the hyper-parameters used to learn the sample weights are the learning rate, the number of epochs, and the coefficients (i.e., *α* and *β*). The ranges of the hyper-parameters are {16, 32, 64, 128} for the hidden nodes, {0.001, 0.01} for the learning rate, and {100, 300, 500} for the epochs. Both *α* and *β* are set from {1, 1e+4, 1e+7, 1e+10}. The effect of hyper-parameter tuning for our method is visualized in Figure S2. All neural-network models, including the auto-encoder for the proposed model, are initialized with a uniform distribution. We use BCELoss as a loss function for classification.

## Data Availability

The data analyzed in this paper are from MarketScan Commercial Claims and Encounters, with more than 100 million patients from 2012 to 2017. Access to the MarketScan data are provided by the Ohio State University. The dataset is available from IBM at MarketScan: https://www.ibm.com/products/marketscan-research-databases. The source code is available from the Github repository at https://github.com/yeon-lab/stable-prediction or the Zenodo repository at https://doi.org/10.5281/zenodo.7826125.

## Supplemental information

Document S1. Tables S1–S6 and Figures S1 and S2

Document S2. Article plus supplemental information
