# Supplementary material for: Stable clinical risk prediction against distribution shift in electronic health records
Source: Patterns (N Y). 2023 Aug 22;4(9):100828. doi: 10.1016/j.patter.2023.100828 (PMC10499849; doi:10.1016/j.patter.2023.100828)
Supplement: Document S1. Tables S1–S6 and Figures S1 and S2 [file mmc1.pdf]

Patterns, Volume *4*

Supplemental information

Stable clinical risk prediction against distribution shift in electronic health records

Seungyeon Lee, Changchang Yin, and Ping Zhang

Supplemental Tables

Supplemental Table S1. The definition of heart failure from EHRs

PMID	26524702, 26687987, 21156884, 15606986
Criteria	ICD-10-CM
Diagnostic codes	I11<br>I13<br>I50<br>I42

Supplemental Table S2. The definition of stroke from EHRs

PMID	29202795
Criteria	ICD-10-CM
Diagnostic codes	Z86,73<br>I60-I69<br>G458<br>G459<br><br>subarachnoid hemorrhage (I60);<br>intracerebral hemorrhage (I61);<br>cerebral infarction (I63);<br>and other transient cerebral ischemic attacks and related syndromes and<br>transient cerebral ischemic attack (unspecified) (G458 and G459),

Supplemental Table S3. Comparison of prediction performance between the full-length medical codes and the shortened codes on the post-shift test set for heart failure and stroke prediction tasks. GRU model is used for the experiments.

		360 days		180 days		90 days	
	Number of letters	AUROC	Accuracy	AUROC	Accuracy	AUROC	Accuracy
HF	5	0.5926 ± 0.006	0.5253 ± 0.006	0.6512 ± 0.008	0.5844 ± 0.005	0.6817 ± 0.006	0.6192 ± 0.006
	4	0.5881 ± 0.013	0.5268 ± 0.007	0.6563 ± 0.003	0.5854 ± 0.006	0.6908 ± 0.006	0.6312 ± 0.005
	3	0.6004 ± 0.009	0.5293 ± 0.006	0.6698 ± 0.002	0.5869 ± 0.004	0.7011 ± 0.002	0.6301 ± 0.004
	2	0.6118 ± 0.010	0.5268 ± 0.005	0.6694 ± 0.001	0.5886 ± 0.003	0.7070 ± 0.003	0.6319 ± 0.003
	1	0.5345 ± 0.005	0.5031 ± 0.011	0.5844 ± 0.003	0.5451 ± 0.004	0.5827 ± 0.006	0.5447 ± 0.003
ST	5	0.5732 ± 0.006	0.5221 ± 0.006	0.5877 ± 0.012	0.5497 ± 0.002	0.6224 ± 0.007	0.5779 ± 0.004
	4	0.5803 ± 0.012	0.5253 ± 0.004	0.5989 ± 0.025	0.5492 ± 0.002	0.6220 ± 0.005	0.5784 ± 0.002
	3	0.5834 ± 0.012	0.5256 ± 0.008	0.6095 ± 0.007	0.5526 ± 0.003	0.6359 ± 0.004	0.5844 ± 0.006
	2	0.5841 ± 0.005	0.5251 ± 0.005	0.6097 ± 0.004	0.5527 ± 0.002	0.6365 ± 0.001	0.5864 ± 0.004
	1	0.5376 ± 0.005	0.5206 ± 0.005	0.5569 ± 0.006	0.5291 ± 0.003	0.5747 ± 0.002	0.5418 ± 0.004

Supplemental Table S4. Comparison of prediction performance on the post-shift test set for heart failure prediction. The baseline and proposed method are denoted by Basic and Weighted, respectively. The average score and standard deviation under 10 trials are reported.

Prediction window		360 days			180 days			90 days		
		AUROC	Precision	Recall	AUROC	Precision	Recall	AUROC	Precision	Recall
LSTM	Basic	0.5859 ± 0.020	0.5771 ± 0.004	0.5301 ± 0.002	0.6615 ± 0.008	0.6586 ± 0.006	0.5840 ± 0.006	0.6902 ± 0.006	0.6654 ± 0.008	0.6247 ± 0.007
	Weighted	0.6061 ± 0.015	0.5797 ± 0.005	0.5319 ± 0.002	0.6863 ± 0.010	0.6718 ± 0.007	0.5859 ± 0.007	0.7091 ± 0.008	0.6701 ± 0.010	0.6344 ± 0.008
GRU	Basic	0.5911 ± 0.021	0.5762 ± 0.004	0.5309 ± 0.002	0.6685 ± 0.004	0.6568 ± 0.005	0.5889 ± 0.005	0.6959 ± 0.004	0.6751 ± 0.004	0.6309 ± 0.004
	Weighted	0.6177 ± 0.019	0.5857 ± 0.006	0.5336 ± 0.003	0.6913 ± 0.005	0.6609 ± 0.006	0.5912 ± 0.004	0.7168 ± 0.006	0.6785 ± 0.006	0.6348 ± 0.005
Dipole	Basic	0.6088 ± 0.003	0.5760 ± 0.004	0.5322 ± 0.002	0.6745 ± 0.002	0.6559 ± 0.004	0.5937 ± 0.004	0.6971 ± 0.001	0.6759 ± 0.004	0.6308 ± 0.002
	Weighted	0.6228 ± 0.004	0.5801 ± 0.006	0.5330 ± 0.002	0.6933 ± 0.004	0.6573 ± 0.007	0.5977 ± 0.005	0.7169 ± 0.004	0.6773 ± 0.004	0.6375 ± 0.003
Retain	Basic	0.6091 ± 0.007	0.5856 ± 0.004	0.5414 ± 0.003	0.6702 ± 0.003	0.6554 ± 0.004	0.5912 ± 0.004	0.7045 ± 0.002	0.6740 ± 0.004	0.6362 ± 0.003
	Weighted	0.6271 ± 0.008	0.5896 ± 0.005	0.5428 ± 0.003	0.6911 ± 0.006	0.6611 ± 0.004	0.5983 ± 0.004	0.7233 ± 0.003	0.6764 ± 0.005	0.6422 ± 0.003
ConCare	Basic	0.6110 ± 0.005	0.5869 ± 0.002	0.5421 ± 0.001	0.6732 ± 0.003	0.6584 ± 0.003	0.5866 ± 0.002	0.7034 ± 0.003	0.6741 ± 0.003	0.6353 ± 0.003
	Weighted	0.6433 ± 0.005	0.5949 ± 0.002	0.5491 ± 0.001	0.6954 ± 0.005	0.6618 ± 0.004	0.5906 ± 0.003	0.7211 ± 0.004	0.6753 ± 0.004	0.6437 ± 0.003
StageNet	Basic	0.6171 ± 0.004	0.5740 ± 0.002	0.5305 ± 0.001	0.6697 ± 0.003	0.6626 ± 0.004	0.5829 ± 0.002	0.6999 ± 0.002	0.6703 ± 0.002	0.6326 ± 0.001
	Weighted	0.6355 ± 0.007	0.5913 ± 0.004	0.5441 ± 0.002	0.6886 ± 0.007	0.6646 ± 0.004	0.5899 ± 0.004	0.7214 ± 0.004	0.6757 ± 0.004	0.6410 ± 0.002

Supplemental Table S5. Comparison of prediction performance on the post-shift test set for stroke prediction. The baseline and proposed method are denoted by Basic and Weighted, respectively. The average score and standard deviation under 10 trials are reported.

Prediction window		360 days			180 days			90 days		
		AUROC	Precision	Recall	AUROC	Precision	Recall	AUROC	Precision	Recall
LSTM	Basic	0.5729 ± 0.015	0.5503 ± 0.005	0.5212 ± 0.003	0.5855 ± 0.014	0.6161 ± 0.006	0.5522 ± 0.002	0.6262 ± 0.008	0.6091 ± 0.013	0.5685 ± 0.008
	Weighted	0.5952 ± 0.019	0.5568 ± 0.006	0.5253 ± 0.004	0.6056 ± 0.017	0.6174 ± 0.007	0.5573 ± 0.003	0.6441 ± 0.011	0.6218 ± 0.013	0.5792 ± 0.009
GRU	Basic	0.5838 ± 0.009	0.5511 ± 0.005	0.5210 ± 0.002	0.6109 ± 0.004	0.6177 ± 0.004	0.5574 ± 0.006	0.6320 ± 0.007	0.6277 ± 0.005	0.5815 ± 0.006
	Weighted	0.6061 ± 0.014	0.5570 ± 0.006	0.5278 ± 0.003	0.6300 ± 0.008	0.6218 ±0.005	0.5608 ± 0.007	0.6485 ± 0.010	0.6288 ± 0.006	0.5843 ± 0.006
Dipole	Basic	0.5862 ± 0.004	0.5617 ± 0.004	0.5275 ± 0.002	0.6141 ± 0.004	0.6153 ± 0.007	0.5592 ± 0.003	0.6340 ± 0.004	0.6278 ± 0.003	0.5827 ± 0.003
	Weighted	0.6050 ± 0.006	0.5625 ± 0.005	0.5290 ± 0.003	0.6318 ± 0.006	0.6159 ± 0.007	0.5601 ± 0.003	0.6494 ± 0.006	0.6333 ± 0.004	0.5920 ± 0.004
Retain	Basic	0.5875 ± 0.006	0.5595 ± 0.005	0.5259 ± 0.003	0.6168 ± 0.005	0.6180 ± 0.004	0.5563 ± 0.004	0.6371 ± 0.003	0.6203 ± 0.006	0.5781 ± 0.003
	Weighted	0.6054 ± 0.007	0.5636 ± 0.005	0.5279 ± 0.002	0.6366 ± 0.008	0.6194 ± 0.005	0.5598 ± 0.005	0.6490 ± 0.004	0.6221 ± 0.007	0.5986 ± 0.003
ConCare	Basic	0.5896 ± 0.006	0.5593 ± 0.007	0.5261 ± 0.005	0.6254 ± 0.003	0.6165 ± 0.004	0.5606 ± 0.003	0.6367 ± 0.004	0.6276 ± 0.005	0.5852 ± 0.002
	Weighted	0.6098 ± 0.009	0.5596 ± 0.007	0.5343 ± 0.005	0.6434 ± 0.007	0.6201 ± 0.005	0.5669 ± 0.003	0.6533 ± 0.006	0.6274 ± 0.006	0.5872 ± 0.003
StageNet	Basic	0.5883 ± 0.007	0.5510 ± 0.001	0.5201 ± 0.001	0.6261 ± 0.006	0.6173 ± 0.004	0.5594 ± 0.004	0.6371 ± 0.003	0.6317 ± 0.005	0.5780 ± 0.002
	Weighted	0.6058 ± 0.010	0.5524 ± 0.002	0.5216 ± 0.002	0.6455 ± 0.007	0.6207 ± 0.004	0.5606 ± 0.005	0.6547 ± 0.007	0.6343 ± 0.006	0.5849 ± 0.003

Supplemental Table S6. Results of the Friedman and Wilcoxon tests on AUPRC scores. The Friedman test is conducted to compare the proposed method with the baselines, including DG and AdaDiag. On the other hand, the Wilcoxon test is applied to compare the proposed method with DG specifically.

	days	p-value (Friedman)	p-value (Wilcoxon)
	360	0.0035	0.0059
HF	180	0.0074	0.0371
	90	0.0018	0.0488
	360	0.0013	0.0019
ST	180	0.0002	0.0273
	90	0.0003	0.0106

Supplemental Figures

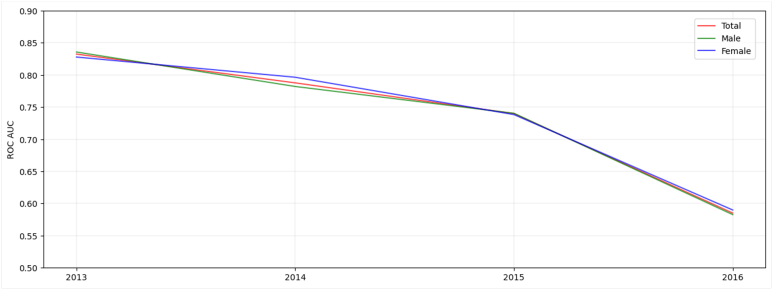

(a) Heart Failur

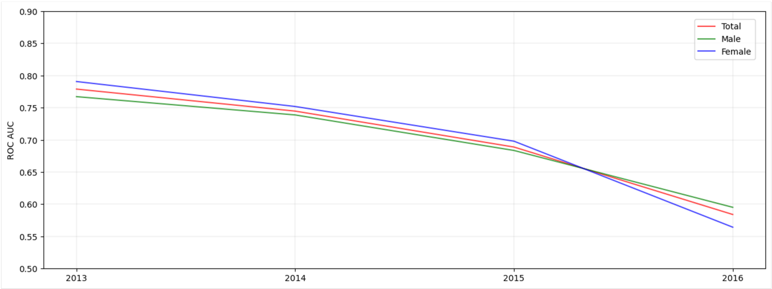

(b) Stroke

Supplemental Figure S1. Visualization of performance per year for heart failure and stoke risk prediction. The x-axis indicates the years and y-axis represents AUROC scores. The AUROC scores are averaged across the entire population as well as separately for male and female patients. The model is based on a GRU architecture and trained only with patients up to 2013.

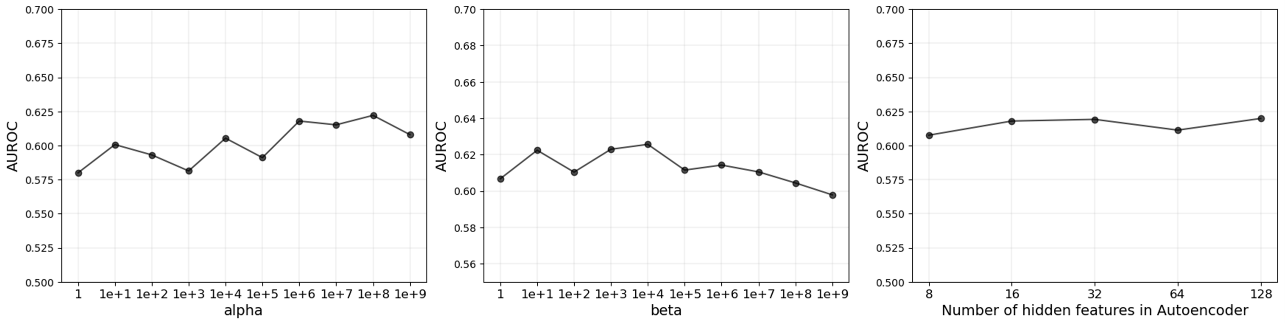

(a) (b) (c)

Supplemental Figure S2. Visualization of effect of hyperparameter tuning on the model performance. The x-axis indicates the values of the hyperparameter and y-axis represents AUROC scores.
